# Real-time guided endodontics versus conventional freehand access cavity preparation by a specialist– an ex vivo comparative study

**DOI:** 10.1007/s00784-025-06310-8

**Published:** 2025-04-09

**Authors:** Hauke Hildebrand, Ralf Krug, Wadim Leontiev, Dorothea Dagassan-Berndt, Gabriel Krastl, Roland Weiger, Thomas Connert

**Affiliations:** 1 Department of Periodontology, Endodontology and Cariology, University Center for Dental Medicine UZB, University of Basel, Basel, Switzerland; 2 Department of Conservative Dentistry and Periodontology, University Hospital of Würzburg, Würzburg, Germany; 3 Dental Imaging, University Center for Dental Medicine Basel UZB, University of Basel, Basel, Switzerland

**Keywords:** Endodontic access cavity, Pulp canal calcification, Pulp canal obliteration, Surgical planning software, Dynamic navigation

## Abstract

**Objectives:**

This study compared conventional access cavity preparation (CONV) and real-time guided endodontics (RTGE) in teeth with pulp canal calcification (PCC) regarding the rate of detected root canals, substance loss and procedural time.

**Materials and methods:**

A total of 72 extracted, sound human teeth (48 incisors, 24 canines) with PCC were matched in pairs, divided into two groups of 36 teeth each. An endodontic specialist performed CONV, while a general dentist not specialized in endodontics utilized RTGE on six models each, under simulated clinical conditions. The operators recorded the time to access the calcified root canals. Pre- and postoperative cone-beam computed tomography (CBCT) scans were obtained to measure substance loss. Statistical significance was tested by examining the overlap of 95% confidence intervals (CIs) and Fisher’s exact test.

**Results:**

RTGE had a marginally higher success rate (34/36) in detecting root canals compared to CONV (32/36) (*p* =.67). While RTGE resulted in less substance loss (CI: 9–14.3 mm^3^ vs. 15–24.2 mm^3^), it required more procedural time than CONV (CI: 11.6–17.8 min vs. 1.4–2.5 min).

**Conclusions:**

Both CONV by a specialist and RTGE by a general dentist achieved a high detection rate of root canals, with RTGE resulting in superior tooth substance preservation but at the expense of longer operation times.

**Clinical relevance:**

RTGE can be considered as an alternative technique for non-specialists when treating teeth with PCC, emphasizing procedural safety and effective canal detection.

## Introduction

In endodontics, dentists encounter challenging clinical situations when treating teeth affected by pulp canal calcification (PCC), leading to reduced treatment success due to technical difficulties [1–5].

The ability to detect calcified root canals depends on the operator’s experience, as shown in trials with 3D-printed teeth and studies comparing practitioners with varying levels of expertise [6, 7]. Therefore, complex cases are commonly referred to a specialist in endodontics [8].

The tools available for conventional access cavity preparation (CONV) have improved in recent decades, for example through optical magnification or the use of cone beam computed tomography (CBCT) [9, 10]. Yet, studies focusing on endodontic treatment of teeth with PCC utilizing modern technical equipment are rare. Kiefner et al. showed success rates up to 90% withCONV in calcified teeth, carried out by a specialized dentist limited to endodontics [11, 12]. However, the observed high success rates should not lead to generalized conclusions, due to the reliance on the individual practitioner’s expertise.

Guided Endodontics (GE) is reported to be a reliable and accurate technique locating calcified root canals. Much like in the field of oral implantology, static navigation followed by dynamic navigation showed promising results when compared with CONV [13, 14]. However, existing studies have primarily focused on the accuracy of the technique and on comparisons with CONV using 3D-printed teeth, which currently lack anatomical features for orientation during CONV [6, 15, 16].

A recent study comparing GE to CONV on human teeth showed that while GE could not outperform an endodontic specialist. But GE enabled a general dentist to operate equivalently, without extensive training [11]. This study highlights the relevance of trials involving human specimens.

There is limited research on comparing performances of CONV with RTGE in an ex vivo setting, with Dianat et al. being the only study to our knowledge [17]. Their focus on procedural accuracy did not fully address substance loss, which is critical for the structural integrity of the tooth [18]. In addition, the specific potential of RTGE for non-specialist dentists was not the subject of the study.

Therefore, the objective of this study is to compare CONV and RTGE using matched extracted human teeth under simulated clinical conditions, focusing on parameters such as the rate of detected root canals, substance loss, procedural time, and additional radiographs when the treatment is carried out by either a specialist or a dentist not specialized in endodontics.

## Materials and methods

### Tooth selection and model fabrication

Seventy-two extracted human calcified teeth (48 incisors, 24 canines), were selected. Teeth were included in the study if the radiologically visible pulp was calcified to at least 2 mm below the deepest part of the cementoenamel junction, as determined using standardized two-dimensional X-rays. The extractions were performed for reasons unrelated to this research and their use was approved by the local research ethics committee (protocol number EKNZ UBE-15/ 111).

The teeth were preserved in 0.1% thymol solution and processed without any association of patient data. They were paired by two independent examiners (WL, TC) according to tooth type, crown and root length, existence of fillings, and degree of calcification as determined by two dimensional radiographs. Tooth length and pulp-incisal distance did not exceed a difference by more than 10% between matched pairs.

The matched tooth pairs were divided into two groups. Supplemented with premolars and molars, maxillary and mandibular study models were produced.

### Surface and CBCT scan

Models were randomly selected for both groups.

Each model was scanned using cone-beam computed tomography (CBCT) (Accuitomo 170; Morita Manufacturing Corp, Kyoto, Japan) at 80 KV and 6 mA using a voxel size of 250 μm and a field of view of 10 × 10 cm. A comprehensive capture of the entire model was obtained through the utilization of a broad field of view, thereby ensuring precise alignment with the subsequent optical scan. For further processing, the CBCT data was exported in DICOM format. A marker tray (DENAMARK & DENATRAY, Mininavident AG, Liestal, Switzerland) was attached in the posterior region of the six models intended for the RTGE Group utilizing a silicone-based impression material (Affinis, Coltene, Alstätten, Switzerland). Surface scans were performed using an intraoral scanner (TRIOS 3 Basic, 3Shape A/S, Copenhagen, Denmark) and saved in STL format.

### Access cavity Preparation (ACP)

In the CONV group, six models were assigned to an endodontic specialist with seven years of experience as a board-certified specialist. Preoperative CBCT data was provided to the operator. Models were placed in a dental simulation unit (Dentsply Sirona, Charlotte, USA) in order to simulate a clinical setting. The specialist performed CONV on the anterior teeth (*n* = 36) using a high-speed contra-angle handpiece with constant water-cooling (1:5, KaVo Master Series; KaVo Dental GmbH, Biberach, Germany) with a cylindrical round-end bur with a diameter of 1.0 mm (837 KR; Intensiv SA, Montagnola, Switzerland), a 1:1 contra-angle handpiece (1:1, KaVo Master Series; KaVo Dental GmbH) with long neck carbide bur (EndoTracer, KaVo Dental GmbH) and diamond coated sonic tips (Sonicflex endo, KaVo Dental GmbH) attached to an airscaler (Sonicflex 2003 L, KaVo GmbH). In all cases, the operator used a surgical microscope (OPMI Proergo; Carl-Zeiss AG, Jena, Germany) to differentiate the different dentin layers. Techniques to detect obliterated root canals, such as the champagne bubble test, were allowed to use the full range of conventional means.

For the RTGE group, the remaining six models were given to a general dentist with five years of professional experience, who was not specialized in endodontics. Planning and treatment were performed by the operator after a training according to the protocol of Leontiev et al. [19].

For digital planning of the ACP, the surface scan and CBCT data were registered in a planning software (coDiagnostiX, Dental Wings Inc., Montreal, Canada). A 3D-model of the marker was loaded into the software and registered to the scanned marker. A true to size virtual drill was placed towards the orifice of each calcified root canal. Case planning was exported in the Generic Planning Objects Container format.

Similar to the CONV group, models were mounted in a dental simulation unit and the marker containing trays were attached to the dental arch. The planning files were uploaded to the dynamic navigation system (DNS) (DENACAM; Mininavident AG, Liestal, Switzerland) and bur registration was performed using the DENAREG tool (Mininavident AG), according to the manufacturers instruction. ACP was performed using similar rotating instruments as in the CONV group. Dynamic navigation was provided to the operator by displaying the real-time position of the bur and its angular and spatial deviation from the preoperative planning, as well as a depth control on a screen next to the operating field. If deemed necessary, the operator of RTGE was also allowed to use an OPMI.

Both operators recorded the procedural time from the start of the clinical treatment until the root canal was accessed and scouted. The successful localization was confirmed with an ISO 10 file (C-Pilot; VDW GmbH, Munich, Germany). A perforation was also defined as failure even when the root canal was finally detected. Both operators were free to acquire additional two-dimensional radiographs during ACP to confirm the preparation axis.

The operators were permitted to stop the procedure based on own their own assessment, if the risk of iatrogenic harm was judged to be excessive. In such instance, the tooth was regarded as a failure and was excluded from further analysis.

### Substance loss measurement and statistical analysis

After ACP, postoperative CBCT scans were acquired for all models with the previously specified settings. Preoperative scans in which each tooth was segmented were matched with the postoperative data using the same threshold. Subtraction of the root canal segmentation (preoperative) from the summation of the root canal and access cavity segmentation (postoperative) resulted in the segmentation of the access cavity. Its volume was then automatically determined using coDiagnostiX software. Teeth in which the root canal could not be detected were excluded from this analysis (*n* = 5).

An independent investigator, who was not involved in the experimental procedures (WL), carried out the pre- and postoperative volume assessments. Microsoft Excel V 16.75 (Microsoft Corporation, Seattle, Washington, USA) and R (R Foundation for Statistical Computing, Vienna, Austria) was used for statistical analysis. The detection rate was evaluated for statistical significance using Fisher’s exact test with the significance level set at *P* <.05. Mean values for the parameters were recorded and differences between groups were tested for significance by examining the overlap of 95% confidence intervals (CIs).

## Results

In CONV, the specialist was able to detect 32 out of 36 root canals, while the general practitioner using RTGE successfully located 34 out of 36. Although RTGE demonstrated a higher success rate, the difference was not significant (*p* =.67). Perforation occurred in one root canal in the CONV group, yet the operator managed to locate the root canal orifice.

**Fig. 1:**
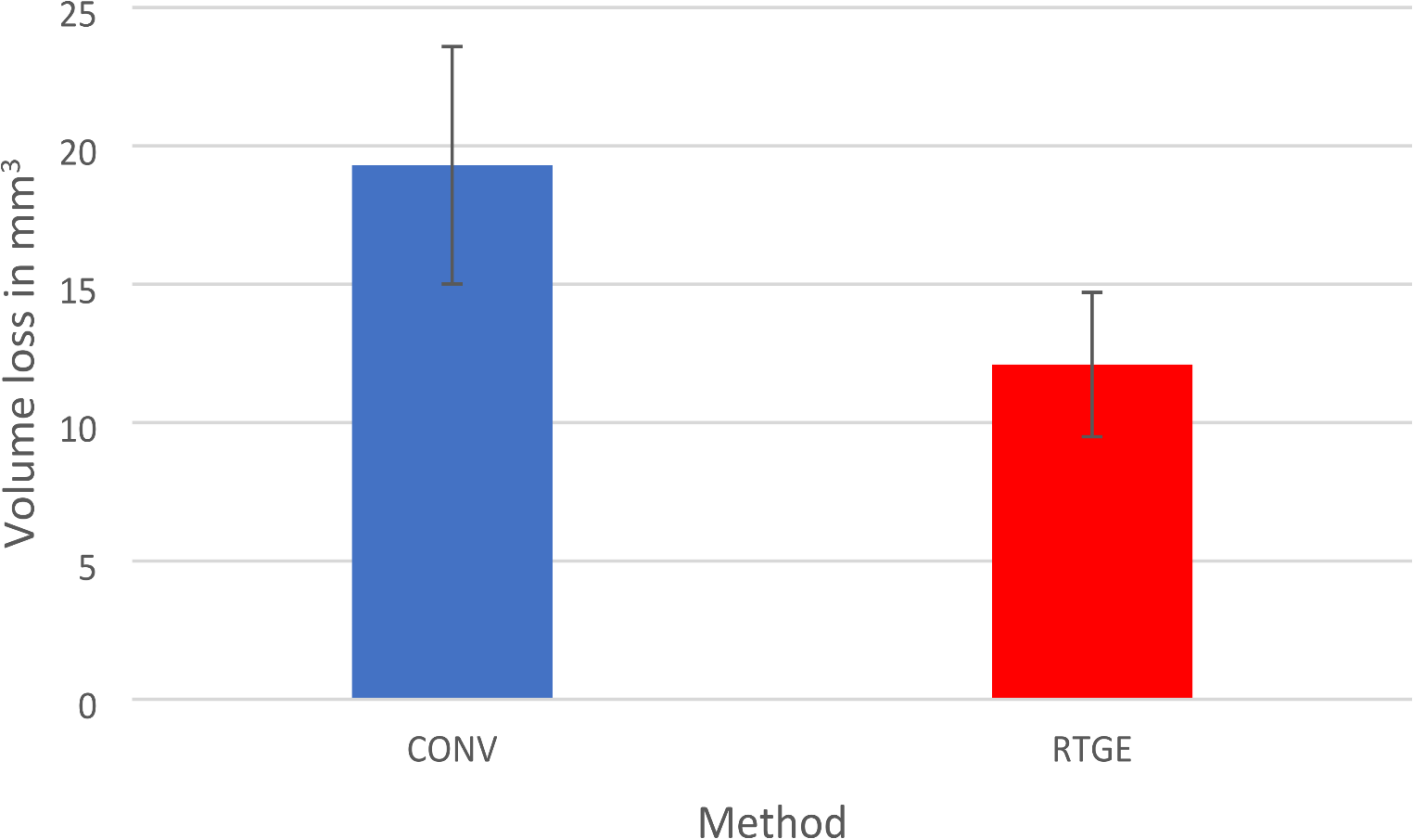
Mean volume of tooth substance loss (mm^3^; with 95% confidence intervals) for the CONV and RTGE

RTGE achieved ACP with significantly less substance loss (mean values: CONV 19.6 mm^3^, CI: 15–24.2 mm^3^ vs. RTGE 11.7 mm^3^, CI: 9–14.3 mm^3^; Fig. 1).

However, in eight cases, CONV resulted in lower substance loss, although the difference was not statistically significant due to overlapping confidence intervals (CONV 9.5 mm³, CI: 4.2–14.9 mm³ vs. RTGE 15.5 mm³, CI: 10.2–20.9 mm³).

**Fig. 2:**
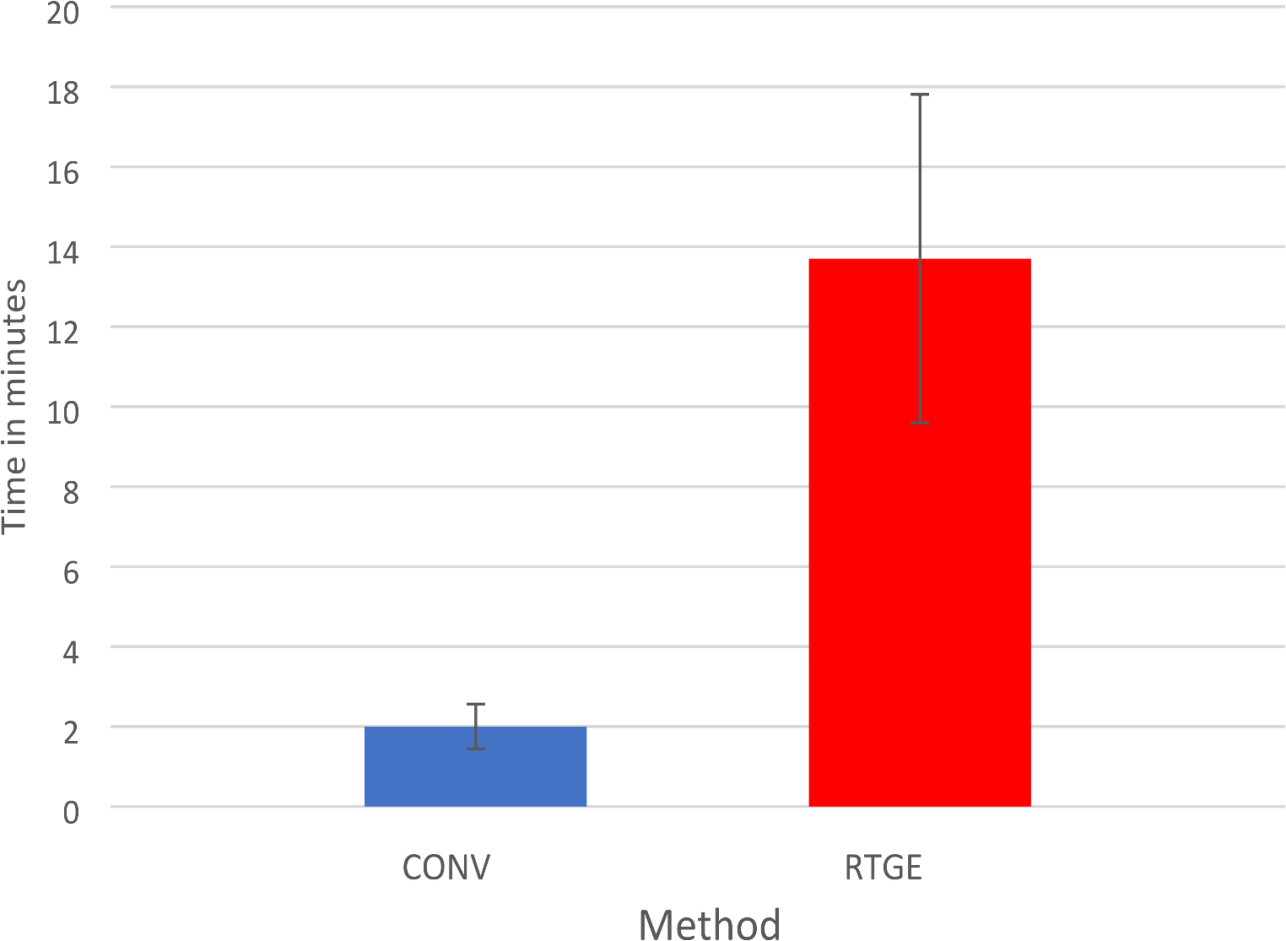
Mean time (min; with 95% confidence intervals) required for root canal detection for CONV and RTGE

There was a significantly shorter procedure time in the CONV group (mean values: CONV 2.0 min, CI: 1.4–2.5 min vs. RTGE 13.7 min, CI: 11.6–17.8 min) (Fig. 2) and required fewer additional radiographs (CONV 7 vs. RTGE 3).

Table 1 provides a summary of the results in terms of root canal detection, mean values for substance loss, procedure time, and additional X-rays for both groups.

**Table 1:** Outcome regarding number of root canals detected (Fisher`s exact test), mean substance loss, procedural time and number of additional periapical radiographs required for CONV versus RTGE (CI: confidence interval); *= not significant

Method (operator)	Detected canals (*n*)	Substance loss (95% CI) [mm^3^]	Procedural time (95% CI) [min]	Additional periapical radiographs (*n*)	Perforations (*n*)
CONV (specialist)	32/36*	19.6 (15.0–24.2)	2.1 (1.4–2.5)	7	1
RTGE (generalist)	34/36*	11.7 (9.0–14.3)	13.7 (11.6–17.8)	3	0

## Discussion

The present study demonstrated the successful application of RTGE by a general practitioner in human teeth with PCC being slightly, but not significant, superior to CONV by a specialist. RTGE provided better results regarding substance loss, despite requiring more operating time. Additionally, less intraoperative X-rays were obtained and no perforations were observed compared to CONV by an endodontic specialist.

In this study, RTGE preserved more tooth substance (CONV 17.6 mm^3^ vs. RTGE 11.7 mm^3^ mean volume loss), potentially enhancing fracture resistance of the treated teeth. The advantage for RTGE found in this study aligns with the recent literature, though this benefit was found to be lower, especially compared to studies using 3D-printed teeth [6, 15, 17].

This might be due to the absence of genuine coloration and the various characteristics of the dentinal hard tissue. This aspect is critical for orientation during ACP, as primary, secondary, and tertiary dentin create a pattern commonly known as a “roadmap”, offering guidance to help to locate the root canal orifice in a calcified tooth. Knowledge of anatomical landmarks and experience in their interpretation are crucial factors in the successful utilization of conventional techniques for managing PCC. Toubes et al. showed that an intraoperative CBCT after ACP can also lead to the successful detection of root canals [20]. Experienced endodontists use optical magnification to differentiate dentin structures in order to localize canal orifices [10, 21].

Consequently, the monochrome resin material in 3D-printed teeth could result in a disadvantage for CONV and makes it not directly transferable to a clinical situation [22].

Dianat et al., as the sole study using human teeth to compare performance on RTGE, determined procedural accuracy by measuring angular deviations and distances from the outer dentin border at the canal entrance and the cementoenamel junction, an area where substance loss can be particularly critical for tooth fracture loads [17, 23].

**Fig. 3:**
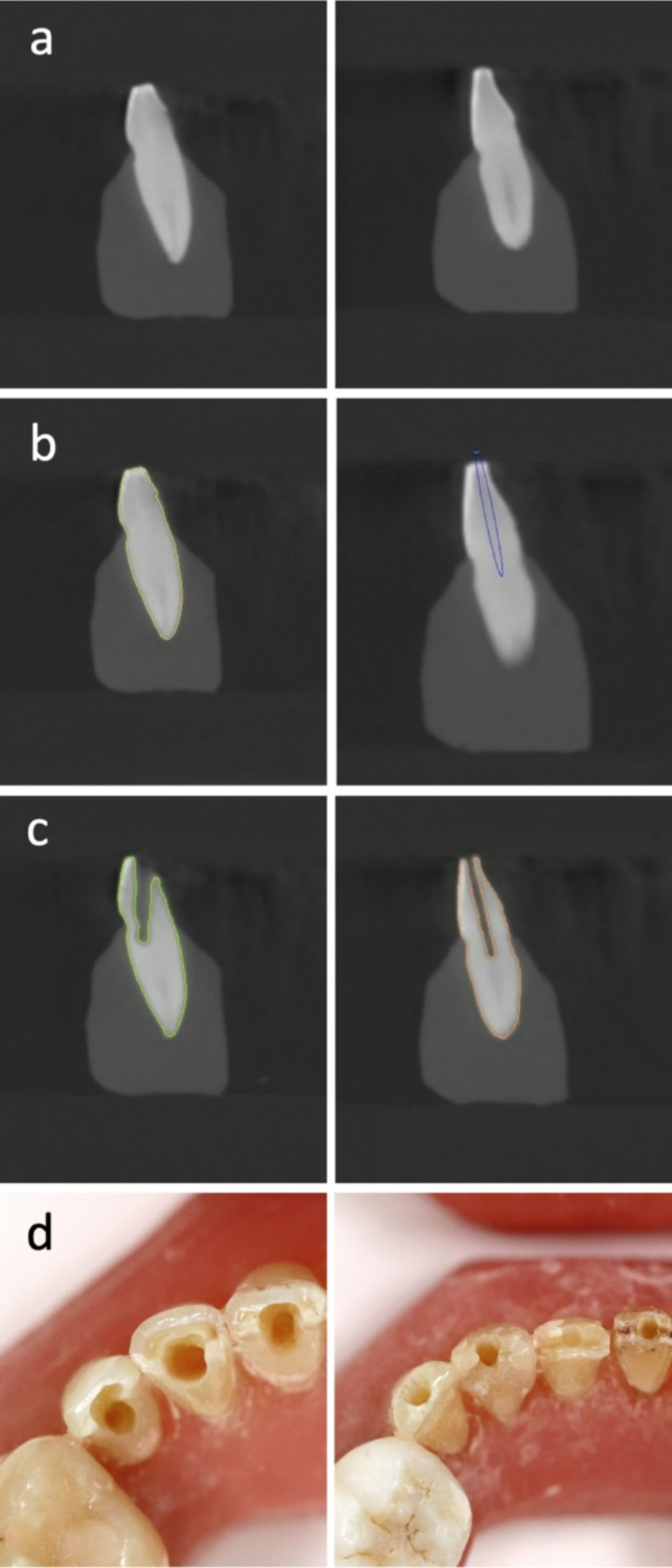
Sagittal view of CBCT data of matched teeth no. 32 from CONV (left) and RTGE (right) model before ACP (**a**), digital ACP planning for RTGE Group (**b**), sagittal view of postoperative CBCT data (**c**) and occlusal view after ACP (**d**)

CONV rarely maintains a straight trajectory (Fig. 3). RTGE, unlike static GE, also allows deviations from a linear drilling axis due to operator inconsistencies. Given the unpredictable nature of dentin loss and the overall difficult assessment for tooth fractures following root canal treatment, definitive conclusions about the impact of different ACPs remain speculative [18, 24–27].

Nevertheless, it seems plausible that preservation of dental hard tissue appears to be generally beneficial for the structural integrity of the tooth. Therefore, this study focused on substance loss.

Treatment times were significantly lower in the CONV group and comparable to those in similar studies involving specialists preparing teeth with PCC [11, 17].

This stands in contrast to other studies comparing both techniques [6, 15, 17].

Dianat et al. compared RTGE with CONV utilized by two operators with different level of experience using human teeth. The findings demonstrated an advantage for RTGE regarding the time needed. However, there was a substantial lower improvement in treatment time when switching between methods for the more experienced operator compared to the inexperienced practitioner (58 vs. 14%) [17].

The small difference in treatment time between CONV and RTGE for specialists and the findings of this study, suggest that the benefit of RTGE for a specialist might not be as substantial as previously anticipated.

Additionally, the less favorable outcome for RTGE regarding operation time could be due to the experience of the practitioner and the presence of more application steps with RTGE, such as calibration after each instrument.

A limitation of this study is the lack of comparability between the individual operators, as only one technique was performed per operator. Research on RTGE and GE shows that the techniques are relatively operator-independent [6, 7, 16]. An individual factor of the operator seems to be primarily crucial for CONV, with distinctive differences in substance loss between specialists and novices [6, 7]. This underlines the assumption that RTGE can not only be highly effective in the procedure according to the results discussed, but may also specifically enable general dentists to achieve a higher level of performance.

Therefore, the work focuses on the capabilities of the RTGE technique compared to an endodontic specialist, in a clinical setup utilizing human teeth to make the environment as realistic as possible.

Due to a are large sample size for both groups and individually precisely matched tooth pairs, bias has been reduced as much as possible.

Another drawback was the influence of individual factors in risk assessment when root canal detection failed. It is important to consider the possibility that other individuals might have achieved greater success with both methods. When compared to findings from studies on related topics, the success rates observed in this investigation were comparable [7, 12, 17]. In a clinical scenario, the dentist could opt for an apicoectomy with retrograde preparation instead of tooth extraction.

The CBCT scan resolution used in this study (250 μm) is relatively low and could be considered a limitation in volumetric analysis. However, De Kinkelder et al. demonstrated that due to motion artifacts in live patients, a resolution below 100 μm is not feasible in a realistic clinical scenario [28]. Furthermore, intergroup comparisons of volume measurements between 125 μm, 200 μm, and 250 μm have shown no significant differences [29]. Given these considerations and the aim to scan the entire model, a lower resolution was deemed acceptable.

RTGE offers several advantages, including reliability, precision and other proven advantages of static navigation, while providing flexibility during ACP and the ability to treat patients with acute symptoms by avoiding the creation of a template [6, 13, 30, 31].

However, disadvantages include considerable costs acquiring a system and its relatively short period of clinical application.

Unlike static GE, where the drill path is constrained by the physical boundaries of a template, RTGE relies on a virtual guided axis, which may be influenced by the operator’s handling.

Studies in implantology have shown that dynamic navigation is subject to a certain learning curve and requires training [32].

In conclusion, both methods demonstrated high treatment success. RTGE showed a higher rate of detected root canals and showed fewer perforations. While volume loss was lower with RTGE, CONV had reduced procedure time. These findings support the assumption that RTGE shows benefits not only for endodontists but in particular for general practitioners for the preparation of access cavities in teeth with PCC.

This approach helps to minimize procedural errors, such as perforations, while preserving tooth structure.

While RTGE has been shown to improve the parameters evaluated, it does not address other aspects of the procedure when treating teeth with PCC. Therefore, this complex treatment should only be carried out by specialists in this field.

Nevertheless, these results are subject to limitations, as this is an ex vivo study. Clinical research is therefore essential to confirm the findings.

## Data Availability

No datasets were generated or analysed during the current study.
